# Validation of Cefiderocol Package Insert Dosing Recommendation for Patients Receiving Continuous Renal Replacement Therapy: A Prospective Multicenter Pharmacokinetic Study

**DOI:** 10.1093/ofid/ofae451

**Published:** 2024-10-21

**Authors:** Aliaa Fouad, Emir Kobic, Nelson P Nicolasora, Melissa L Thompson Bastin, Paul M Adams, Yuwei Shen, Andrew J Fratoni, Xiaoyi Ye, Joseph L Kuti, David P Nicolau, Tomefa E Asempa

**Affiliations:** Center for Anti-Infective Research and Development, Hartford Hospital, Hartford, Connecticut, USA; Department of Pharmacy, Banner–University Medical Center, Phoenix, Arizona, USA; Division of Infectious Diseases, Banner–University Medical Center, Phoenix, Arizona, USA; Department of Pharmacy Services, University of Kentucky Medical Center, Lexington, Kentucky, USA; Department of Pharmacy Practice and Science, University of Kentucky College of Pharmacy, Lexington, Kentucky, USA; Division of Nephrology, Bone and Mineral Metabolism, Department of Internal Medicine, University of Kentucky College of Medicine, Lexington, Kentucky, USA; Center for Anti-Infective Research and Development, Hartford Hospital, Hartford, Connecticut, USA; Center for Anti-Infective Research and Development, Hartford Hospital, Hartford, Connecticut, USA; Division of Nephrology, Hartford Hospital, Hartford, Connecticut, USA; Center for Anti-Infective Research and Development, Hartford Hospital, Hartford, Connecticut, USA; Center for Anti-Infective Research and Development, Hartford Hospital, Hartford, Connecticut, USA; Division of Infectious Diseases, Hartford Hospital, Hartford, Connecticut, USA; Center for Anti-Infective Research and Development, Hartford Hospital, Hartford, Connecticut, USA

**Keywords:** AKI, cefiderocol, CRRT, effluent flow rate, transmembrane clearance

## Abstract

**Background:**

Cefiderocol is the first antibiotic with effluent flow rate–based dosing recommendations outlined in the product label for patients receiving continuous renal replacement therapy (CRRT). We aimed to investigate the population pharmacokinetics of cefiderocol among patients receiving CRRT and validate these dosing recommendations.

**Methods:**

A multicenter, prospective cefiderocol pharmacokinetic study among intensive care unit patients receiving CRRT was conducted (2022–2023). Blood sampling was performed at steady-state and cefiderocol concentrations were assayed by validated liquid chromatography–tandem mass spectrometry. Population pharmacokinetic analyses were conducted in Pmetrics using R software. The free time above the minimum inhibitory concentration (*f* T > MIC) and total daily area under the concentration time curve (AUC_daily_) were calculated.

**Results:**

Fourteen patients with effluent flow rates ranging from 2.1 to 5.1 L/h were enrolled. Cefiderocol concentrations best fitted a 2-compartment model. Mean ± standard deviation (SD) parameter estimates for clearance, central compartment volume, and intercompartment transfer constants (k_12_ and k_21_) were 3.5 ± 1.5 L/hour, 10.7 ± 8.4 L, 3.9 ± 1.8 hours^−1^, and 2.2 ± 2.2 hours^−1^, respectively. With simulations based on product label dosing recommendations, all patients achieved 100% *f*T > MIC up to MIC 8 mg/L with an AUC_daily_ (mean ± SD) of 1444 ± 423 mg × hour/L. Cefiderocol was well tolerated among the 14 patients.

**Conclusions:**

The current package insert dosing recommendations resulted in pharmacodynamically optimized cefiderocol exposures. Cefiderocol concentrations exceeded relevant MIC breakpoints in all patients at each effluent flow rate, and AUC_daily_ was within the range observed in patients in the phase 3 clinical trials, suggestive of a safe and therapeutic drug profile.

Acute kidney injury (AKI) is a common complication of critical illness and is defined as a rapid increase in serum creatinine, decrease in urine output, or both [1–3]. Continuous renal replacement therapy (CRRT) is the preferred renal support modality in critically ill patients with AKI, with nearly 1 in 5 patients with sepsis-associated AKI in the hospital requiring renal support [4–6]. CRRT includes multiple modalities such as continuous venovenous hemofiltration (CVVH), continuous venovenous hemodialysis (CVVHD), and continuous venovenous hemodiafiltration (CVVHDF) and allows for correction of uremia, electrolyte, and acid-base disorders as well as gradual solute and fluid removal [5, 6].

The use of CRRT support in critically ill patients creates a dynamic process for drug removal, with several variables that can affect the pharmacokinetics of drugs and subsequently alter the rate of drug clearance [7–9]. For instance, antibiotics that have low protein binding are more readily removed with CRRT [8]. Conversely, antibiotics with a large volume of distribution into tissue are less efficiently removed by CRRT [8]. Mechanical factors of CRRT can also affect drug clearance. For example, transmembrane pressure can be altered by increasing the dialysate or blood flow rate, thereby impacting drug clearance in CRRT [10]. Finally, the size of the filter membrane pores is directly proportional to the degree of drug removal by CRRT [7–9].

Cefiderocol is a siderophore cephalosporin antibiotic approved for the treatment of complicated urinary tract infections (cUTIs) and nosocomial pneumonia (hospital-acquired bacterial pneumonia [HABP]/ventilator-associated bacterial pneumonia [VABP]), which are both frequently treated infections in critically ill patients [11–13]. The elimination of cefiderocol is primarily through glomerular filtration with approximately 99% excreted in the urine [12]. As a result, dosing must be modified in patients across the spectrum of renal function (ie, creatinine clearance [CrCl]). Notably, cefiderocol is also the first antibiotic with United States Food and Drug Administration (FDA)–approved dosing recommendations based on a patient's CRRT effluent flow rate [12]. These dosing recommendations were derived from resimulation of a limited number of patients (n = 9) who were receiving CRRT during the cefiderocol development program as well as in vitro modeling demonstrating predictable transmembrane clearance (CL_TM_) through contemporary CRRT filters [12, 14–16].

More robust data are needed to validate this novel dosing recommendation based on a range of effluent flow rates. To that end, the goal of this pragmatic multicenter study was to explore the population pharmacokinetics of cefiderocol among critically ill patients receiving CRRT and validate the CRRT-based dosing recommendations as outlined in the product label.

## MATERIALS AND METHODS

### Study Design

This was a prospective, multicenter, open-label, pharmacokinetic (PK) study of cefiderocol in critically ill adult patients receiving CRRT support (ClinicalTrials.gov identifier NCT05373615). Patient enrollment occurred at 3 participating sites: Hartford Hospital (Hartford, Connecticut), Banner–University Medical Center (Phoenix, Arizona), and University of Kentucky Medical Center (Lexington, Kentucky). The study protocol and informed consent documents were reviewed and approved by each local institutional review board (see the Supplementary Materials for more information). Written informed consent was obtained from patient, legal authorized representative, or next of kin, according to local regulations. All PK and statistical analyses were performed at the Center for Anti-Infective Research and Development (CAIRD) at Hartford Hospital. This study was conducted in accordance with principles derived from international guidelines including the Declaration of Helsinki and applicable International Council on Harmonisation of Technical Requirements for Pharmaceuticals for Human Use Good Clinical Practice guidelines.

### Participants

Fourteen critically ill patients were enrolled in the study. This study was open to CRRT patients receiving cefiderocol to treat actual or suspected infections, as well as CRRT patients who received cefiderocol for the sole purpose of PK blood sampling. Full inclusion and exclusion criteria are detailed in Supplementary Table 1.

Screening and baseline assessments and procedures were completed within 24 hours before the start of the study drug infusion. For each patient, a complete medical and surgical history was obtained and a physical examination performed. Chemistry profile, blood count, and urinalysis were assessed in screening evaluations. Acute Physiology and Chronic Health Evaluation (APACHE) II assessment was calculated at baseline. CRRT settings and all prior medications taken or received within 3 days before study drug infusion were recorded.

### Antibiotic Dose and Administration

Cefiderocol was prepared according to the product label and administered intravenously. Per FDA product labeling, the recommended CRRT dosing regimen of cefiderocol is 1.5 g every 12 hours (q12h), 2 g q12h, 1.5 g every 8 hours (q8h), and 2 g q8h as a 3-hour infusion based on effluent flow rates of ≤2 L/hour, 2.1 to 3 L/hour, 3.1 to 4 L/hour, and ≥4.1 L/hour, respectively (Supplementary Table 2) [12]. Local prescribers were also permitted to use an alternative dose based on individual patient factors such as residual renal function, obesity, etc.

### Sample Collection and Processing

Blood samples to assess cefiderocol concentrations were collected in 4 mL dipotassium ethylenediaminetetraacetic acid (K_2_EDTA) vacutainer tubes at 0 hours, before the first dose of cefiderocol was administered. Remaining prefilter samples were collected after a minimum of 2 doses as follows: 0 hours (just prior to the start of the dose), 1.5 hours (halfway through the 3-hour cefiderocol infusion), 3 hours (peak), and 4, 6, 8, and 12 hours (only in 12-hour regimens) after the start of the final dose. Additional postfilter samples were collected at 4 hours and 8 hours to assess filter clearance. All blood samples were centrifuged within 30 minutes of collection under refrigeration (2°C–8°C) at 1500*g* for 10 minutes. Plasma was separated into cryovials and frozen at −80° C until concentration analysis. A dialysate sample was obtained from the effluent waste bag at the 6-hour time point and frozen at −80°C until concentration analysis. For patients with a urine output of ≥100 mL/day 24 hours prior to last dose, an additional serum creatinine was collected within 30 minutes prior to the start of the final dose to evaluate the residual renal function and CrCl (a scenario whereby the kidneys of a patient on CRRT still produce some urine and potentially contribute to additional solute and drug clearance in parallel with CRRT).

CrCl was calculated using the following equation:


$$\mathrm{Measured}\;\mathrm{CrCL}\;(\mathrm{mL}/\min)=\frac{\mathrm{UCr}\;\left(\frac{\mathrm{mg}}{\mathrm{dL}}\right)*\mathrm{urine}\;\mathrm{volume}\;(\mathrm{mL})}{\mathrm{Predose}\;\mathrm{SCr}\;\left(\frac{\mathrm{mg}}{\mathrm{dL}}\right)*\mathrm{time}\;(\min)}$$

where CrCl is creatinine clearance, SCr is serum creatinine, UCr is urine creatinine, and time is the number of minutes of the urine collection interval.

An additional 2 K_2_EDTA tubes were collected prefilter for cefiderocol protein binding determination at the 3-hour time point. Plasma was separated by centrifugation as described above. Then, 0.9 mL of plasma was pipetted into an ultrafiltration device (Centrifree, Merck Millipore Ltd, Ireland) in triplicate and centrifuged under refrigeration (2°C–8°C) at 1500*g* for 45 minutes to obtain the protein-free filtrate. Ultrafiltrate cryovials was stored at −80°C until concentration determination. The free (protein unbound) fraction was calculated by dividing the ultrafiltrate concentration by the total plasma concentration.

Cefiderocol concentrations in plasma, ultrafiltrate, and dialysate during the clinical study were determined by a validated ultra-high-performance liquid chromatography–tandem mass spectrometry by CAIRD. Additional details of the assay are provided in the Supplementary Materials.

### Saturation Coefficient

To assess filter clearance (ie, saturation coefficient [SA]), prefilter blood samples at 4, 6, and 8 hours were collected. Postfilter blood samples were collected at the 4-hour and 8-hour time points and processed as described above. SA was calculated using the following equation:


$$SA=\frac{2*C_{\mathrm{dialysate}}}{C_{\mathrm{pre}}+\;C_{\mathrm{post}}}$$

where C_dialysate_ is the cefiderocol concentration from the effluent waste bag at 6 hours, C_pre_ is the cefiderocol concentration at the 6-hour prefilter time point, and C_post_ is the mean percent removal at 4 and 8 hours [17].

### Transmembrane Clearance

Clearance by CRRT was estimated using the SA and ultrafiltrate rate (Q_uf_). A dilutional correction factor was incorporated into the clearance equation to account for the prefilter replacement fluid utilizing the following equation:


$$CL_{TM}=\frac{\left(SA*Q_{uf}*Q_{b}\right)}{\left(\mathrm{Qb}+Q_{\mathrm{rep}}\right)}$$

where Q_b_ is the blood flow rate and Q_rep_ is the prefilter replacement fluid rate [17].

### Pharmacokinetic Analyses

Cefiderocol prefilter concentrations were fitted to 1- or 2-compartment models using the nonparametric adaptive grid algorithm in the Pmetrics package (Laboratory of Applied Pharmacokinetics, Los Angeles, California) for R software. A multiplicative assay variance model was determined by fitting a polynomial to the plot of the interday assay standard deviation (SD) versus the measured cefiderocol concentrations, respectively, generating the following formulas: SD = γ × (0.006 + 0.06 × C), where C is cefiderocol concentration and γ is the proportional error model (ie, environmental noise). Final model selection was determined based on the Akaike information criterion (AIC) value, visual inspection of the observed versus predicted plots, and assessment of PK parameter estimates for each participant with resimulation of each patient's concentration time curve for visual inspection. After selection of a base model, linear regression (GraphPad Prism software version 10.0.3, La Jolla, California) was used to evaluate relationships between PK parameters and effluent rates, CL_TM_, and body weight.

### Pharmacodynamic Analyses

Using each patient's individual PK parameters, a simulation of cefiderocol exposure based on the dosing regimen recommended by the product label according to their effluent flow rate was performed. The derived free cefiderocol concentration profile of each patient was assessed for free percent time above the minimum inhibitory concentration (%*f*T > MIC) with target of ≥75% *f*T > MIC associated with clinical success [14, 18]. *f*T > MIC exposure was assessed over a range of MICs including the cefiderocol FDA and Clinical and Laboratory Standards Institute (CLSI) susceptibility breakpoints for gram-negatives [19]. Steady-state total drug area under the concentration time curve (AUC) was calculated as the recommended daily doses divided by the Bayesian individual clearance. The daily AUC (AUC_daily_) values were compared with the mean AUC_daily_ values achieved in the phase 3 cefiderocol clinical trials, which excluded patients supported on CRRT, as a surrogate for safety and tolerability [12, 20].

### Safety Assessment

Participants were monitored for any sign or symptom of adverse events (AEs) throughout the course of the study after signing of informed consent. All AEs requiring medical attention were treated by the local study physician and were recorded by the investigator.

## RESULTS

### Patient and CRRT Characteristics

Fourteen critically ill patients on CVVHDF renal support (Prismaflex 7.2 control unit, Baxter Healthcare Corporation) were enrolled from August 2022 until December 2023; 7 at Hartford Hospital, 5 at Banner–University Medical Center, and 2 at University of Kentucky Medical Center. Patient demographics, cefiderocol dosing, and CRRT characteristics are presented in Table 1. The average effluent dose was 32.7 mL/kg/hour and effluent flow rates ranged from 2.08 to 5.06 L/hour. Five patients were dosed as per product label, and 9 patients had additional dose adjustments due to obesity, residual renal function, or effluent flow rates that fluctuated between dosing cutoffs.

**Table 1.** Patient and Continuous Renal Replacement Therapy Characteristics for Enrolled Patients Receiving Cefiderocol

Characteristic	Total (N = 14)
Demographics	
Age, y, mean ± SD (range)	55.4 ± 14.9 (33–77)
Male sex, No. (%)	9 (64.3)
Body weight, kg, mean ± SD (range)	113.5 ± 57.3 (62.4–265)
BMI, kg/m^2^, mean ± SD (range)	37.6 ± 16.2 (21–73)
APACHE II score, mean ± SD (range)	19 ± 6.24 (10–32)
Baseline hematocrit, %, mean ± SD (range)	26.8 ± 3 (21.8–32.5)
Baseline albumin, g/dL, mean ± SD (range)	3 ± 0.6 (1.6–3.8)
Cefiderocol dosing	
Dosage regimen received, No. (%)	
2 g q6h	5 (35.7)
2 g q8h	1 (7.1)
1.5 g q8h	3 (21.4)
2 g q12h	4 (28.6)
1.5 g q12h	1 (7.1)
Patients on cefiderocol for actual or suspected infection, No. (%)	5 (35.7)
CRRT characteristics	
Effluent dose, mL/kg/h, mean ± SD (range)	32.7 ± 10 (18–60)
Effluent flow rate, L/h, mean ± SD (range)	3.34 ± 0.9 (2.08–5.1)
Effluent flow rate, No. (%)	
≤2 L/h	1 (7.1)
2.1–3 L/h	4 (28.6)
3.1–4 L/h	7 (50)
≥4.1 L/h	2 (14.3)
CRRT CVVHDF mode	14 (100)
Filter type, No. (%)	
M100	9 (64.3)
HF1400	2 (14.3)
ST100	3 (21.4)

Abbreviations: APACHE, Acute Physiology and Chronic Health Evaluation; BMI, body mass index; CRRT, continuous renal replacement therapy; CVVHDF, continuous venovenous hemodiafiltration; q6h, every 6 hours; q8h, every 8 hours; q12h, every 12 hours; SD, standard deviation.

### Saturation Coefficient and Transmembrane Clearance

The mean ± SD of SA was 0.8 ± 0.6 (range, 0.34–1.1). The SA mean ± SD for M100 filter (n = 9), HF1400 (n = 2), and ST100 (n = 3) filters were 0.8 ± 0.2, 0.6 ± 0.3, and 0.8 ± 0.1, respectively. The CL_TM_ mean ± SD was 2.3 ± 0.6 (range, 1–3.5) L/hour.

### Pharmacokinetics

**Figure 1.**
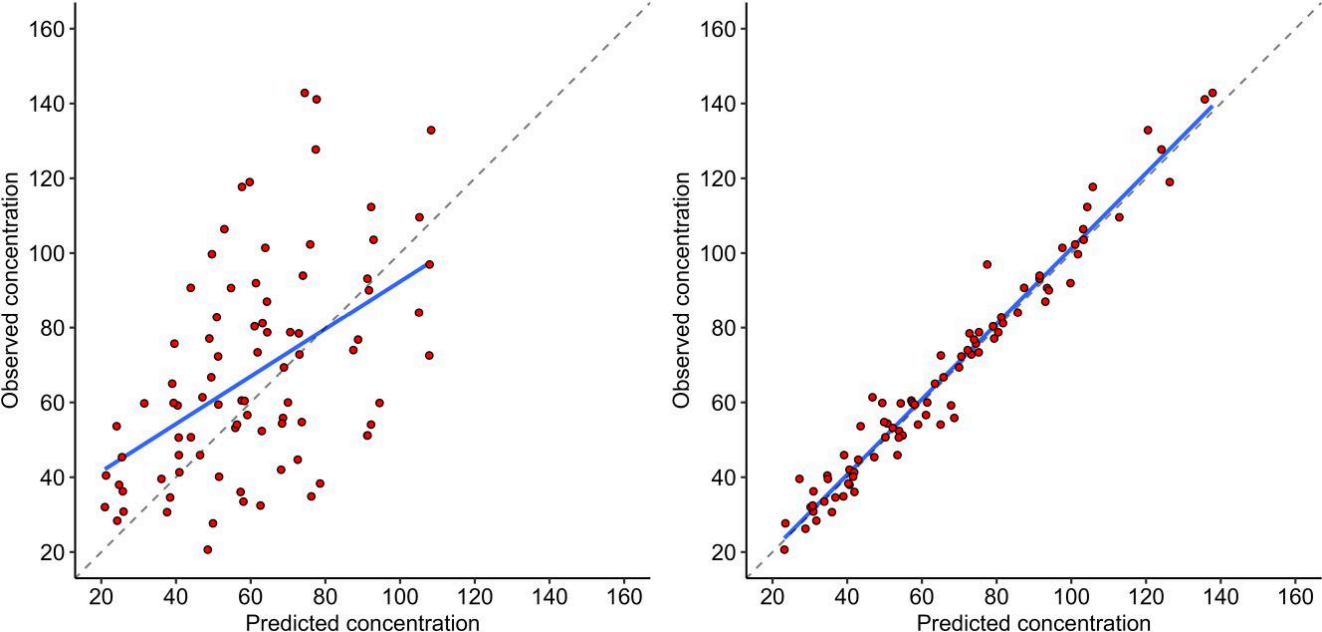
Observed versus population-predicted (left) and maximum a posteriori–Bayesian individual predicted (right) cefiderocol prefilter concentrations (mg/L) for the final 2-compartment model.

A 2-compartment model fitted the data better than a 1-compartment model (AIC, 625.3 vs 656.9). There was a statistically significant relationship between cefiderocol clearance and patient's body weight, effluent rate, and CL_TM_ (Supplementary Figure 1). However, these covariates did not improve AIC from the base model. Final cefiderocol PK parameters from the base model are listed in Table 2. Shrinkage was acceptable for all parameters with values between 0.27 and 5.1. The population-predicted versus observed and maximum a posteriori-Bayesian individual predicted versus observed plots demonstrated good fits (Figure 1). Gamma for the final model was 1.6. Individual patient fits based on the derived individual PK parameters with observed concentration overlaid are presented in Supplementary Figure 2.

**Table 2.** Cefiderocol Population Pharmacokinetic Parameters Derived From 14 Critically Ill Patients on Continuous Renal Replacement Therapy Support

Parameter	Mean ± SD	Median	Shrinkage, %
CL, L/h	3.5 ± 1.46	3.3	0.27
V_c_, L	10.7 ± 8.4	7.4	1.5
k_12_, h^−1^	3.9 ± 1.8	3.5	0.7
k_21_, h^−1^	2.2 ± 2.2	1.5	5.1

Abbreviations: CL, total body clearance; k_12_, intercompartment transfer constant; k_21_, intercompartment transfer constant; SD, standard deviation; V_c_, volume of central compartment.

### Cefiderocol Exposures

**Figure 2.**
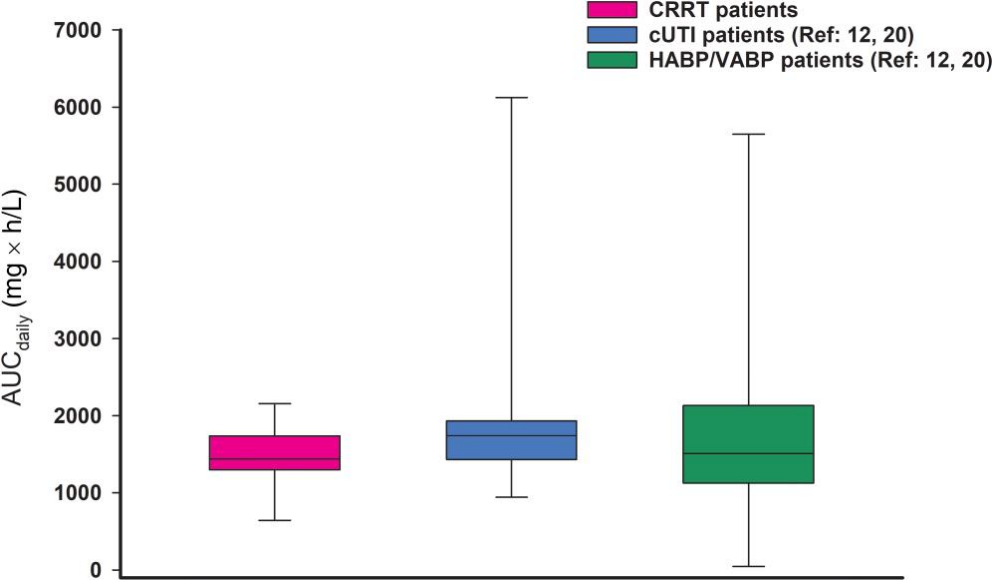
Daily area under the concentration time curve (AUC_daily_) comparison of continuous renal replacement therapy (CRRT) patients (n = 14) to patients in the phase 3 clinical trials (complicated urinary tract infection [cUTI], n = 21; hospital-acquired bacterial pneumonia/ventilator-associated bacterial pneumonia [HABP/VABP], n = 146). Data are presented as median with interquartile range, and whiskers represent the minimum and maximum values for each cohort.

The mean ± SD free fraction of cefiderocol in plasma was 0.84 ± 0.1 (range, 0.69–0.98). Free drug concentration time curves were resimulated for the 9 patients who did not receive cefiderocol dosing according to the package insert. Package insert recommended dosing for cefiderocol per effluent flow rate resulted in high free drug exposures in these critically ill patients, with an exposure of 100% *f*T > MIC achieved up to MIC of 8 mg/L for all patients (Table 3). The study population AUC_daily_ (mean **±** SD) of 1444 ± 423 mg × hour/L was within the range of the drug exposures observed in the phase 3 cUTI and HABP/VABP clinical trials (Figure 2) [12, 20].

**Table 3.** Simulated Cefiderocol Exposures, and Free Time Above the Minimum Inhibitory Concentration for Individual Study Patients

Patient ID	MIC, mg/L						
	0.5	1	2	4	8	16	32
*f*T > MIC, %							
Pt 1^a^	100	100	100	100	100	100	94
Pt 2	100	100	100	100	100	100	100
Pt 3	100	100	100	100	100	100	92
Pt 4	100	100	100	100	100	100	75
Pt 5	100	100	100	100	100	100	100
Pt 6^a^	100	100	100	100	100	100	86
Pt 7	100	100	100	100	100	100	94
Pt 8	100	100	100	100	100	82	0
Pt 9	100	100	100	100	100	100	49
Pt 10^a^	100	100	100	100	100	100	100
Pt 11^a^	100	100	100	100	100	100	100
Pt 12^a^	100	100	100	100	100	100	99
Pt 13	100	100	100	100	100	100	100
Pt 14	100	100	100	100	100	100	6

Abbreviations: *f*T > MIC, free time above the minimum inhibitory concentration; MIC, minimum inhibitory concentration; Pt, patient.

^a^Patients received the package insert–recommended dose for continuous renal replacement therapy.

A comparison of the observed *f*T > MIC and AUC_daily_ exposures for the 9 patients receiving doses outside of package insert with the resimulated package insert dosage is provided in Supplementary Table 3. These data suggest that higher doses were not needed for obese patients or the few with residual renal function.

### Safety Assessments

Cefiderocol was well tolerated, with no serious AEs reported among these critically ill cohort. Four patients experienced 6 mild and 2 moderate AEs, with none affecting a patient's participation in the study. One patient experienced hyponatremia, while diarrhea was also reported for another patient. The third patient experienced moderate confusion (which required medical intervention), elevations of liver function enzymes, and leukocytosis. Mild thrombocytopenia was identified in the fourth patient.

## DISCUSSION

The results of the current study showed that a 2-compartment PK model best described cefiderocol concentrations in this patient population. The final model had low shrinkage values for all PK parameter estimates, indicating reliable individual fits for the study cohort. The total body clearance (3.5 L/hour) and volume of the central compartment (10.7 L) for patients receiving CRRT in this study was comparable to that of patients not requiring CRRT in phase 3 clinical trials (clearance, 4.0 L/hour; volume, 7.8 L) [12, 20]. Despite protein-binding assays not being performed as part of routine clinical care, this was an important analysis to undertake in this current study, as protein binding in critically ill patients varies and CRRT primarily filters free drug [21, 22]. In this study, the fraction of protein-bound and unbound cefiderocol was obtained for each patient, and the PK/pharmacodynamic (PD) assessments conducted with free drug concentrations. We found that cefiderocol average protein binding in the current study (10%–20%) was lower than the average derived from product label or from non–critically ill patients (40%–60%), which may be attributed to the critical illness and hypoalbuminemia of our study population [12].

The PK/PD index of cefiderocol is the %*f*T > MIC, with a target of ≥75% *f*T > MIC predictive of clinical success [14, 18]. Cefiderocol is excreted via the kidneys; thus, its total clearance depends on renal function [23]. Therefore, a dose adjustment based on renal function is recommended to ensure that appropriate cefiderocol exposures can be achieved in all patients across the renal function spectrum including those on CRRT support [14, 24, 25]. This PK study provides data to support the current product label recommendations by demonstrating that cefiderocol drug exposures among patients on CRRT were comparable to exposures in patients with normal renal function. Indeed, using the recommended CRRT dosing, we observed high cefiderocol exposures in all patients at the highest CLSI and FDA susceptible breakpoint of MIC 4 mg/L (ie, 100% *f*T > MIC_4mg/L_) and in the majority of patients up to an MIC of 16 mg/L (ie, >75% *f*T > MIC_16mg/L_). Furthermore, the comparable mean AUC_daily_ between this study cohort and that achieved in cUTI and HABP/VABP clinical trials is suggestive of a similarly safe and efficacious exposure profile among CRRT patients, and further corroborates findings from several published case reports [20, 26–30]. In additional sensitivity analysis, a fixed protein binding of 50% that is associated with non–critically ill patients was applied to all CRRT patient simulations in this study and the resulting cefiderocol exposures were still sufficient at all relevant MICs including 100% *f*T > MIC_4mg/L_.

The importance of optimizing antibiotic doses across the renal function spectrum including CRRT cannot be overstated, as observed with drugs such as ceftolozane/tazobactam, ceftazidime/avibactam, and telavancin that resulted in lower treatment success among patients with renal impairment and patients receiving CRRT, with suggestions that dosing in these populations was not optimized during drug development [31–33]. As such, the inclusion of CRRT dosing recommendations in the cefiderocol product label, and subsequent validation in this study, provides clinicians a simple tool to allow a usually understudied cohort of patients (ie, CRRT patients) access to a therapeutic option for serious bacterial infections.

The multicenter design of this study allowed for assessment of a variety of effluent flow rates as well as filter types leading to a more robust dataset. In addition, the determination of concentrations in postfilter blood samples as well as concentrations in the effluent waste bag allowed us to assess filter clearance for each patient. Of note, the product label also indicates that the recommended dosing regimen may need to be tailored based on residual kidney function and the patient's clinical status. As such, the clinical team of 3 patients administered a higher cefiderocol dose based on effluent flow rate and morbid obesity. Cefiderocol exposures based on the actual doses received as well as the PK simulation with cefiderocol doses that would have been administered if only effluent flow rates were considered were similar (Supplementary Table 3). Subsequently, we found that body weight inclusion in the PK model did not enhance the model fit. This is not surprising, given that the calculation of effluent flow rate incorporates body weight; that is, effluent flow rate (L/hour) = effluent dose (L/kg/hour) × body weight (kg). This suggests that escalating the dosing regimen solely based on obesity is not warranted.

It is important to note limitations of the current study. Among CRRT patients, the package insert recommends drug dosing based on effluent flow rate, which can be calculated with parameters from CVVH, CVVHD, and CVVHDF [12]. However, in this pragmatic trial, only patients supported by CVVHDF mode were encountered but our study results corroborate previous findings that dosing based on effluent rate should apply regardless of the CRRT modality [14]. Future studies with non-CRRT dialysis modalities such as slow low-efficiency dialysis are warranted. Unsurprisingly, the majority of patients enrolled had effluent flow rates between 2 and 4 L/hour; thus, additional studies are required to understand cefiderocol PK at much lower or higher flow rates. In addition, the low number of CRRT units with HF1400 and ST100 filters precludes any sort of comparison of SA between the filters.

Of note, future studies are still needed to address the impact of varying degrees of residual renal function on drug exposure. Importantly, the degree of residual renal function and urine output would influence whether to maintain the dose or escalate to a higher dosing regimen or frequency [26–28]. Finally, more data are also needed in the clinical scenario where CRRT effluent flow rates are changing often (typically during the first 24 hours of CRRT initiation). To optimize clinical success in both these cases, dose escalation may be warranted [34].

## CONCLUSIONS

The FDA-recommended dosing regimen for cefiderocol in patients receiving CRRT resulted in appropriate drug exposures, with concentrations exceeding relevant MIC susceptible breakpoints in all patients at each effluent flow rate. The CRRT study population AUC_daily_ was also within range of the AUC observed in phase 3 clinical trials for patients not requiring CRRT, suggestive of a safe and therapeutic profile.

## Supplementary Data

Supplementary materials are available at *Open Forum Infectious Diseases* online. Consisting of data provided by the authors to benefit the reader, the posted materials are not copyedited and are the sole responsibility of the authors, so questions or comments should be addressed to the corresponding author.

## Supplementary Material

ofae451_Supplementary_Data

## Notes

***Acknowledgments.*** We would like to acknowledge Hanna Roenfanz, Elizabeth Cyr, and Keelyn Kotecki from the Center for Anti-infective Research and Development (Hartford Hospital) and Dr Grace Korkames from Banner–University Medical Center for their assistance in the conduct of this study.

***Disclaimer.*** The funder did not exercise control over the conduct, data analysis, or reporting of the research.

***Financial support*.** The study was funded by Shionogi & Co, Ltd, Japan.

***Potential conflicts of interest*.** E. K. has served as a speaker's bureau member for Shionogi. M. L. T. B. has served on the speaker's bureau and/or advisory board for Baxter Healthcare and Fresenius. J. L. K. has served as a consultant or speaker's bureau member or has received research funding from AbbVie, bioMériuex, Entasis, the US Food and Drug Administration (FDA), Merck, Pfizer, Shionogi, and Venatorx. D. P. N. has served as a consultant or speaker's bureau member or has received research funding from AbbVie, Cepheid, Merck, Pfizer, Shionogi, and Venatorx. T. E. A. has received research funding from Venatorx, Paratek, Shionogi, Spero Therapeutics, and the FDA. All other authors report no potential conflicts.
