# Supplementary material for: Validation of Cefiderocol Package Insert Dosing Recommendation for Patients Receiving Continuous Renal Replacement Therapy: A Prospective Multicenter Pharmacokinetic Study
Source: Open Forum Infect Dis. 2024 Oct 21;11(10):ofae451. doi: 10.1093/ofid/ofae451 (PMC11492798; doi:10.1093/ofid/ofae451)

Supplemental Data – July 18, 2024

**Validation of Cefiderocol Package Insert Dosing Recommendation for Patients receiving Continuous Renal Replacement Therapy: A Prospective Multi-Center Pharmacokinetic Study**

**Aliaa Fouad, PhD^1^, Emir Kobic,** **PharmD, BCIDP^2^, Nelson P. Nicolasora, MD^3^, Melissa L. Thompson Bastin, PharmD, PhD, BCCCP, FCCM^4,5^, Paul M. Adams, MD^6^, Yuwei Shen, PharmD^1^, Andrew J. Fratoni, PharmD^1^, Xiaoyi Ye, MD^7^, Joseph L. Kuti, PharmD, FIDP, FCCP^1^, David P. Nicolau, PharmD, FCCP, FIDSA^1,8^, and Tomefa E. Asempa, PharmD^1*^**

^1^Center for Anti-Infective Research and Development, Hartford Hospital, Hartford, CT, USA

^2^Department of Pharmacy, Banner University Medical Center, Phoenix, AZ, USA

^3^Division of Infectious Diseases, Banner University Medical Center, Phoenix, AZ, USA

^4^Department of Pharmacy Services, University of Kentucky Medical Center, Lexington, KY, USA

^5^Department of Pharmacy Practice and Science, University of Kentucky College of Pharmacy, Lexington, KY, USA

^6^Division of Nephrology, Bone & Mineral Metabolism, Department of Internal Medicine, University of Kentucky College of Medicine, Lexington, KY, USA.

^7^Division of Nephrology, Hartford Hospital, Hartford, CT, USA

^8^Division of Infectious Diseases, Hartford Hospital, Hartford, CT, USA

**Table of Contents**

**Institutional Review Board information 2**

**Table S13**

**Table S24**

**Bioanalytical procedures 5**

**Table S36**

**Figure S17**

**Figure S28**

**Institutional Review Board information:**

**Hartford Hospital**

**IRB board name:** Hartford Hospital Institutional Review Board

**Approval number:** HHC-2022-0045

**Approval date:** 04/27/2022

**Study title**: Effect of Continuous Renal Replacement Therapy and Residual Renal Clearance on Cefiderocol Pharmacokinetics in Critically Ill Adult Patients

**Banner-University Medical Center**

**IRB board name:** University of Arizona Institutional Review Board

**Approval number:** STUDY00001724

**Approval date:** 3/14/2023

**Study title**: Effect of Continuous Renal Replacement Therapy and Residual Renal Clearance on Cefiderocol Pharmacokinetics in Critically Ill Adult Patients

**University of Kentucky Medical Center**

**IRB board name:** University of Kentucky Medical Institutional Review Board

**Approval number:** 78385

**Approval date:** 7/11/2022

**Study title**: Effect of Continuous Renal Replacement Therapy and Residual Renal Clearance on Cefiderocol Pharmacokinetics in Critically Ill Adult Patients

**Table S1:** Inclusion and exclusion criteria for study patients

| Inclusion Criteria | Exclusion Criteria |
| --- | --- |
| - Age 18 years or older | - Pregnant or breast-feeding Females |
| - Receiving CRRT including CVVH, CVVHD, and CVVHDF support | - History of any moderate or severe hypersensitivity or allergic reaction to any β-lactam antibiotic (a history of mild rash to a cephalosporin followed by uneventful re-exposure is not a contraindication) |
|  | - A hemoglobin less than 8 gm/dL at baseline^1^ |
|  | - Acute liver injury, defined as aspartate aminotransferase (AST) or alanine aminotransferase (ALT) > 5 times the upper limit of normal, or AST or ALT > 3 times the upper limit of normal with an associated total bilirubin > 2 times upper limit of normal^1^ |
|  | - Currently receiving or planned to start extracorporeal membrane oxygenation (ECMO) during the conduct of the study |
|  | - Any rapidly-progressing disease or immediately life-threatening illness (defined as imminent death within 48 hours in the opinion of the investigator |

^1^Patients receiving cefiderocol for therapeutic reasons i.e., to treat actual or suspected infections were exempt from this exclusion criteria

| **Effluent flow rate** | **Recommended cefiderocol dosage** |
| --- | --- |
| 2 L/h or less | 1.5 grams every 12 hours as a 3h infusion |
| 2.1 to 3 L/h | 2 grams every 12 hours as a 3h infusion |
| 3.1 to 4 L/h | 1.5 grams every 8 hours as a 3h infusion |
| 4.1 L/h or greater | 2 grams every 8 hours as a 3h infusion |

**Table S2: Recommended dosage of cefiderocol for patients receiving CRRT**

CRRT: continuous renal replacement therapy

**Bioanalytical procedures:** Concentrations of cefiderocol in protein free filtrate and human plasma were determined with validated UPLC methods in saline (0.05 to 100 μg/mL) and K2EDTA plasma 0.1 to 100 μg/mL) using cefiderocol-d8 as the internal standard. The internal standard was diluted with 9:1 acetonitrile/water to yield a 100 ng/mL solution of cefiderocol-d8 for protein precipitation. To a microcentrifuge tube containing 50 μL standard or sample was added 250 μL protein precipitation solution. The suspension was vortexed to mix and centrifuged at 15,000xg for two minutes. For analysis, 50 μL of supernatant transferred to a 96 well plate and diluted with 200 μL water. The sample manager was cooled to 5 °C. Cefiderocol was analyzed using a Waters Acquity UPLC H-Class system with tandem TQ-XS mass spectrometer (LC-MS/MS) equipped with an Acquity UPLC BEH C18 (1.7 μm, 2.1 x 50 mm) column maintained at 40 °C. Samples above the limit of quantification were diluted with blank matrix prior to sample preparation. All compounds were monitored using an ESI probe in positive acquisition mode. The quantitative and qualitative mass transitions for cefiderocol were 752.2 🡪 285.0 and 752.2 🡪 214.1, respectively. The quantitative mass transition for cefiderocol-d8 was 760.3 🡪 293.0. Mean interday coefficients of variance (CV) for low and high values of cefiderocol in saline were 2.7%, 3.5%, and 3.5%, respectively. Cross-matrix validation in K2EDTA human plasma was performed prior to sample analysis. Mean interday CV for low, medium, and high values of cefiderocol in K2EDTA human plasma were 8.3%, 3.2%, and 3.9%, respectively.

| Patient ID | Effluent flow rate (L/h) | Actual cefiderocol dose received | Actual AUC_daily_ (mg*h/L) | Actual free % T>MIC, mg/L | | | | | | | Reason for selection of actual cefiderocol dose per clinical team |
| --- | --- | --- | --- | --- | --- | --- | --- | --- | --- | --- | --- |
|  |  | Simulated cefiderocol dose^#^ | Simulated AUC_daily_ (mg*h/L) | Simulated free % T>MIC, mg/L | | | | | | |  |
|  |  |  |  | **0.5** | **1** | **2** | **4** | **8** | **16** | **32** |  |
| PT 2 | **2.08** | **1.5g q12h** | **1617** | **100%** | **100%** | **100%** | **100%** | **100%** | **100%** | **89%** | Effluent flow rate borderline between 2 dosing cut-offs, thus dose was reduced |
|  |  | 2g q12h | 2156 | 100% | 100% | 100% | 100% | 100% | 100% | 100% |  |
| PT 3 | **2.14** | **2g q12h** | **1966** | **100%** | **100%** | **100%** | **100%** | **100%** | **100%** | **100%** | Effluent flow rate borderline between 2 dosing cut-offs, thus dose was increased |
|  |  | 1.5g q12h | 1474 | 100% | 100% | 100% | 100% | 100% | 100% | 92% |  |
| PT 4 | **3.67** | **2g q6h** | **2494** | **100%** | **100%** | **100%** | **100%** | **100%** | **100%** | **100%** | Patient had urine output of 250 mL in the 24 hours prior to study thus was prescribed a 2g q6h regimen to account for residual renal function. However, during last dose (sampling interval) of the 3 prescribed doses, the patient produced no urine (anuric) but initial dosing regimen was maintained |
|  |  | 1.5g q8h | 1403 | 100% | 100% | 100% | 100% | 100% | 100% | 75% |  |
| PT 5 | **3.41** | **2g q6h** | **2482** | **100%** | **100%** | **100%** | **100%** | **100%** | **100%** | **100%** | Patient had urine output of 580 mL in the 24 hours prior to study and a residual renal function CrCl of 0.5 L/hr thus was prescribed 2g q6h regimen to account for residual renal function |
|  |  | 1.5g q8h | 1396 | 100% | 100% | 100% | 100% | 100% | 100% | 95% |  |
| PT 7 | **4.77** | **2g q6h** | **2086** | **100%** | **100%** | **100%** | **100%** | **100%** | **100%** | **100%** | Dose escalated due to patient’s high BMI (73 kg/m^2^) |
|  |  | 2g q8h | 1565 | 100% | 100% | 100% | 100% | 100% | 100% | 94% |  |
| PT 8 | **3.34** | **2g q8h** | **859** | **100%** | **100%** | **100%** | **100%** | **100%** | **100%** | **26%** | Dose escalated due to patient’s high BMI (57.9 kg/m^2^) |
|  |  | 1.5g q8h | 644 | 100% | 100% | 100% | 100% | 100% | 82% | 0% |  |
| PT 9 | **5.06** | **2g q6h** | **1376** | **100%** | **100%** | **100%** | **100%** | **100%** | **100%** | **82%** | Dose escalated due to patient’s high BMI (45.9 kg/m^2^) |
|  |  | 2g q8h | 1032 | 100% | 100% | 100% | 100% | 100% | 100% | 49% |  |
| PT 13 | **3.02** | **2g q6h** | **1628** | **100%** | **100%** | **100%** | **100%** | **100%** | **100%** | **100%** | Patient had urine output of 2.5 L in the 24 hours prior to study and a residual renal function of CrCL of 0.7 L/hr thus was prescribed 2g q6h regimen to account for residual renal function |
|  |  | 2g q12h | 814 | 100% | 100% | 100% | 100% | 100% | 90% | 33% |  |
| PT 14 | **3.15** | **2g q12h** | **1182** | **100%** | **100%** | **100%** | **100%** | **100%** | **100%** | **61%** | Per clinical team |
|  |  | 1.5g q8h | 1329 | 100% | 100% | 100% | 100% | 100% | 100% | 6% |  |

**Table S3.** Pharmacodynamic indices comparison between the actual administered dosing regimen and the simulated dosing regimen using individual patient’s pharmacokinetics parameters for the nine patients who received a different regimen than the package insert

**^#^**Based on the package insert dosing recommendation: 1.5g q12h, 2g q12h, 1.5g q8h and 2g q8h based on effluent flow rates of ≤2 L/h, 2.1 to 3 L/h, 3.1 to 4 L/h, and ≥4.1 L/h respectively.

**Figure S1.** Linear regression for cefiderocol PK parameters A) Cefiderocol clearance (CL); B) Cefiderocol volume of the central compartment (V_c_) versus body weight, CL_TM_, and Effluent rate


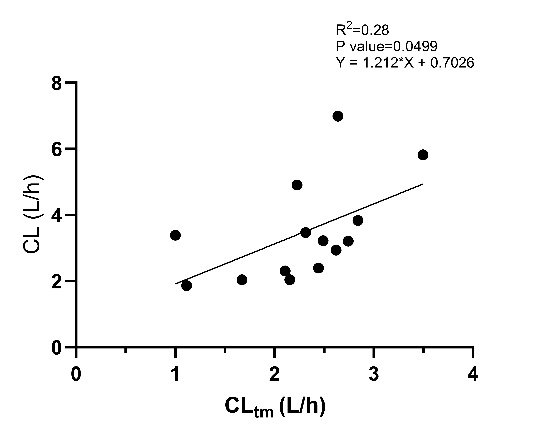


**A**


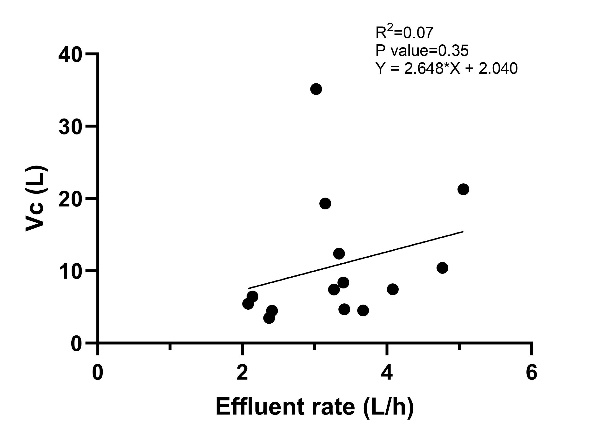

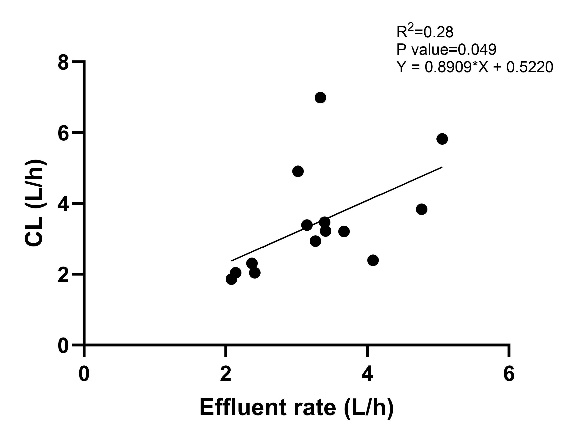

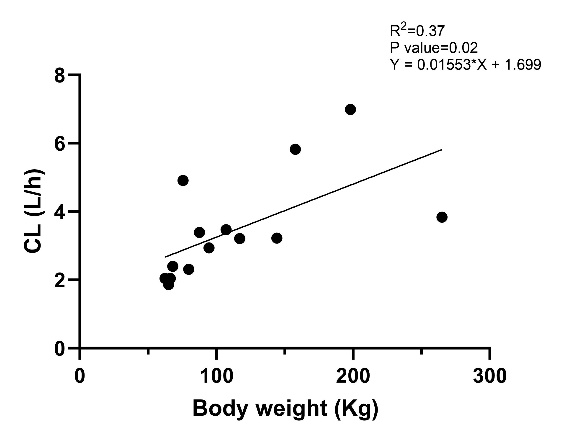


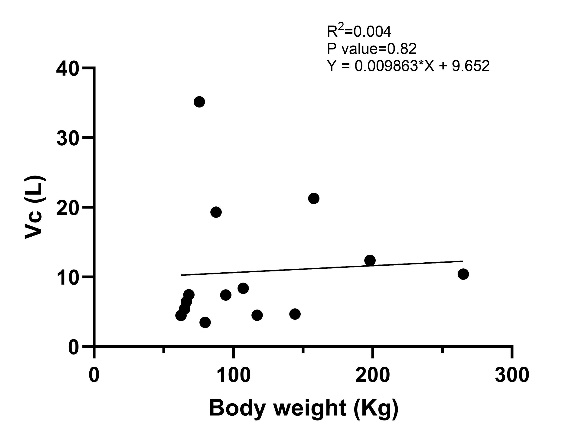

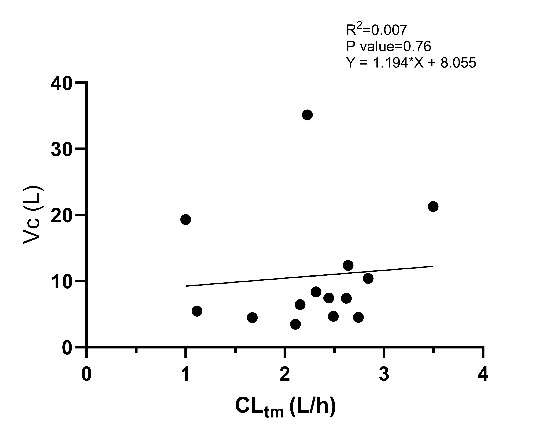


**B**

**Figure S2.** Visual predictive check. Individual observed cefiderocol (FDC) concentrations versus simulated concentration time curves for dosing regimens received during the pharmacokinetic study for the 14 critically ill patients on CRRT


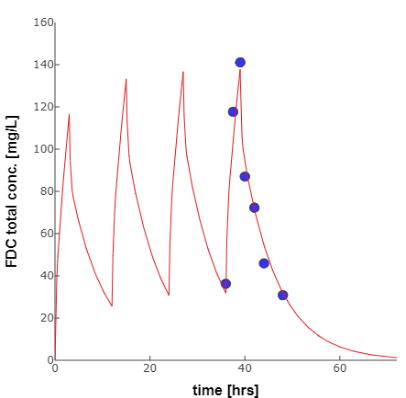

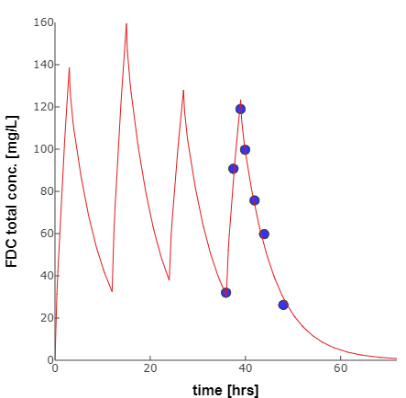

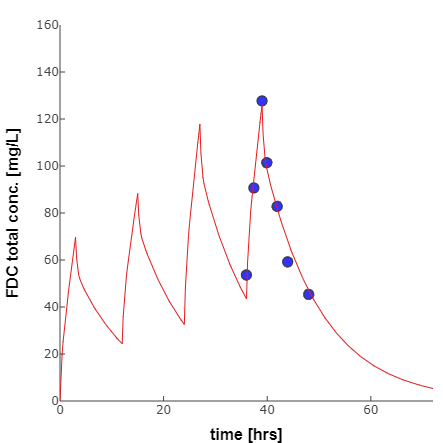

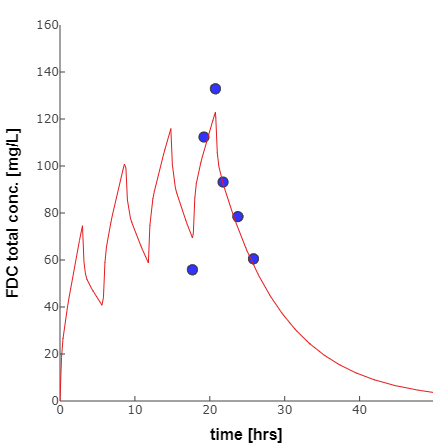

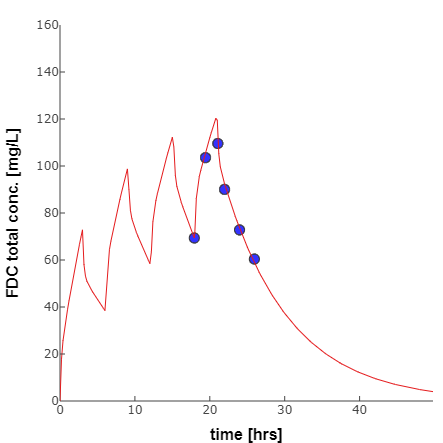

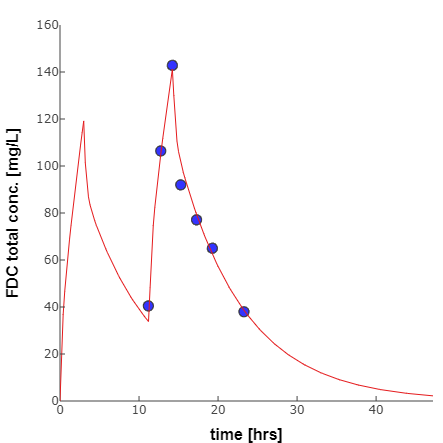

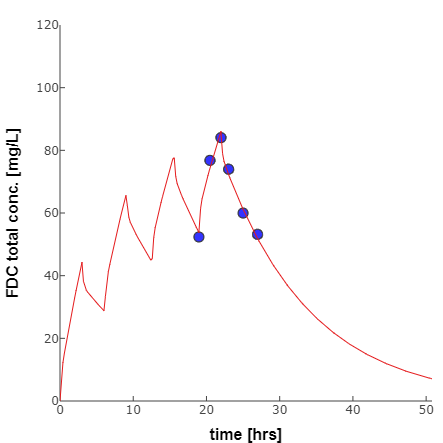

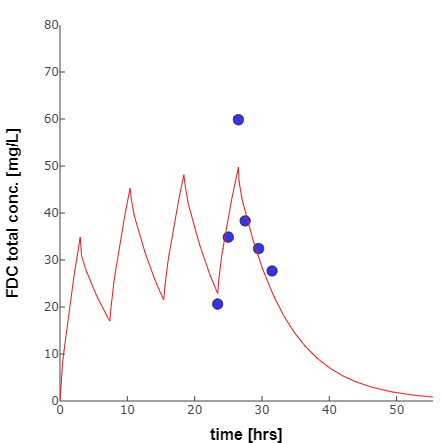

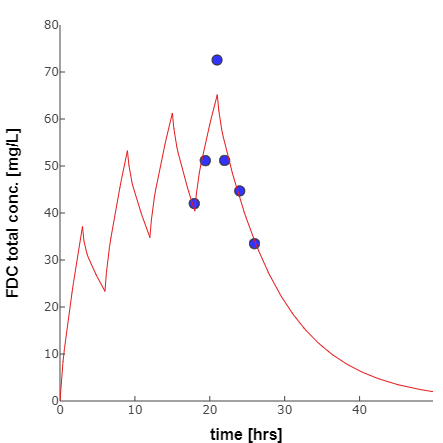

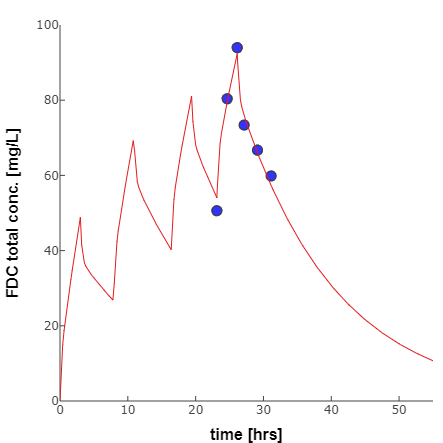

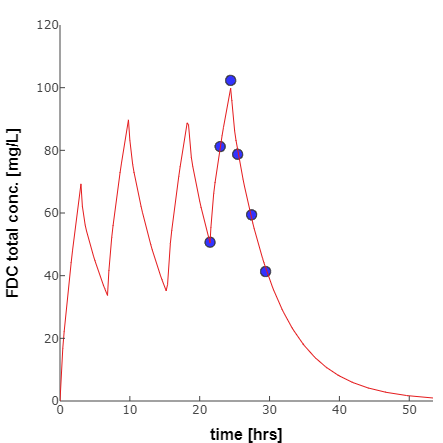

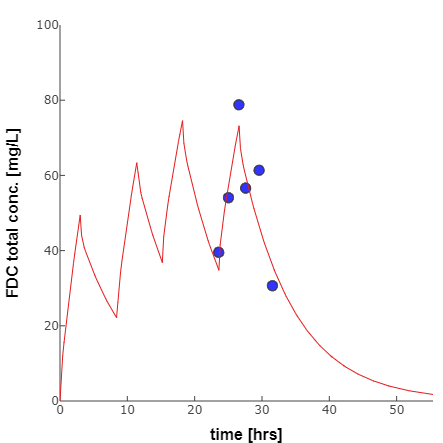

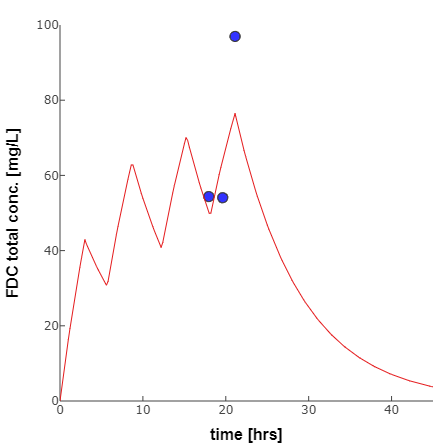

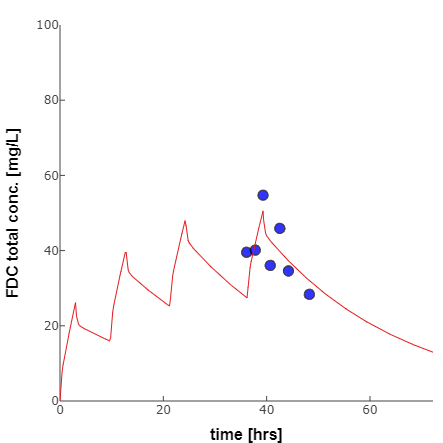

Supplement: ofae451_Supplementary_Data [file ofae451_supplementary_data.docx]
